# Development of an optimised key worker framework for people with dementia, their family and caring unit living in the community

**DOI:** 10.1186/s12913-017-2448-0

**Published:** 2017-07-20

**Authors:** Emma Renehan, Dianne Goeman, Susan Koch

**Affiliations:** 1 RDNS Institute, Royal District Nursing Service Ltd, 31 Alma Rd, St Kilda, Vic 3182 Australia; 2 Central Clinical School, Medicine, Nursing & Health Sciences, Monash University, Melbourne, Australia; 3 School of Medicine & Public Health, The University of Newcastle, Callaghan, Australia

**Keywords:** Dementia, Framework, Key worker, Support worker, Case management, Workforce, People with dementia, Carers, Co-design, Community

## Abstract

**Background:**

In Australia, dementia is a national health priority. With the rising number of people living with dementia and shortage of formal and informal carers predicted in the near future, developing approaches to coordinating services in quality-focused ways is considered an urgent priority. Key worker support models are one approach that have been used to assist people living with dementia and their caring unit coordinate services and navigate service systems; however, there is limited literature outlining comprehensive frameworks for the implementation of community dementia key worker roles in practice. In this paper an optimised key worker framework for people with dementia, their family and caring unit living in the community is developed and presented.

**Methods:**

A number of processes were undertaken to inform the development of a co-designed optimised key worker framework: an expert working and reference group; a systematic review of the literature; and a qualitative evaluation of 14 dementia key worker models operating in Australia involving 14 interviews with organisation managers, 19 with key workers and 15 with people living with dementia and/or their caring unit. Data from the systematic review and evaluation of dementia key worker models were analysed by the researchers and the expert working and reference group using a constant comparative approach to define the essential components of the optimised framework.

**Results:**

The developed framework consisted of four main components: overarching philosophies; organisational context; role definition; and key worker competencies. A number of more clearly defined sub-themes sat under each component. Reflected in the framework is the complexity of the dementia journey and the difficulty in trying to develop a ‘one size fits all’ approach.

**Conclusions:**

This co-designed study led to the development of an evidence based framework which outlines a comprehensive synthesis of components viewed as being essential to the implementation of a dementia key worker model of care in the community. The framework was informed and endorsed by people living with dementia and their caring unit, key workers, managers, Australian industry experts, policy makers and researchers. An evaluation of its effectiveness and relevance for practice within the dementia care space is required.

**Electronic supplementary material:**

The online version of this article (doi:10.1186/s12913-017-2448-0) contains supplementary material, which is available to authorized users.

## Background

Dementia is an umbrella term used to describe a collection of symptoms caused by disorders affecting the brain. It is characterised by disturbance of multiple brain functions, including memory, thinking, orientation, comprehension, calculation, learning capacity, language, judgement, behaviour and the ability to perform everyday tasks [1]. Dementia is a major health problem worldwide impacting on the quality of life and physical, mental, emotional and social health of people living with dementia and their wider family and caring unit. Living with a diagnosis of dementia as well as caring for a person with dementia can lead to a decline in physical and mental health and also impact on employment and education prospects, finances and participation in social and community life [2].

In Australia, dementia is a national health priority. It is the second leading cause of death and the single greatest cause of disability in older people (aged 65 years or older) [3]. Currently, 1.2 million people are estimated to be involved in the care of a person with dementia [4]. There are also more than 353,800 Australians living with dementia with this number expected to increase to 400,000 in the next 5 years [3]. By the 2060s, spending on dementia is set to exceed that of any other health condition, with a projected cost of $83 billion a year [5]. In Australia a large number of people with dementia (70%) live at home with the majority of support provided by informal carers [3] With the rising number of people living with dementia and a shortage of more than 150,000 formal and informal carers expected by 2029 [6] developing approaches to coordinating services in cost-efficient and quality-focused ways is considered an urgent priority.

Health care workers in support roles is one approach that has been used to assist people living with dementia and their caring unit coordinate services and navigate service systems. The titles for such support roles vary worldwide and include: key workers; support workers; case managers; care coordinators; dementia advisers; and Admiral Nurses. For the purposes of this paper the term key worker will be used as it was deemed the most acceptable and appropriate by people living with dementia and carers of people living with dementia who participated in this project. A key worker is a support person who can act as a bridge between professionals and service users, offering practical assistance in fulfilling goals, and performing a range of tasks including healthcare, community participation, assistance in rehabilitation and advocacy to name a few [7].

A key worker role is important as inefficient and fragmented health services can cause major problems for people living with dementia and their caring unit [8, 9] impacting on their physical, emotional and social health. Research has also shown, that despite the availability of support services, uptake is low particularly among carers of people living with dementia [10]. Reasons for this are complex and include issues such as: perceived lack of need; reluctance to accept help; lack of knowledge; accessibility issues; carer identity barriers; fear of role change; and concerns for financial or privacy factors [11–13]. Frustration and confusion with complex health and social care systems and not knowing who to contact have also been associated with low service use [14, 15]. Contact with key worker type roles has been associated with increased service use amongst carers of people living with dementia, indicating that a central contact point within the system is important for linkage into and navigation of support services [11, 14].

Several systematic reviews investigating the impact of dementia key worker roles have been undertaken. These reviews have primarily focused on case management models [16–23] with only one extending this to include integrated care and consumer-directed care models [24]. These reviews have investigated the role’s impact on: health care costs and resource utilisation [16]; general wellbeing [17]; clinical outcomes, satisfaction and service use for people living with dementia and their caring unit [18, 24]; risk of long-term care placement [19]; clinical outcomes and utilisation of resources [20]; its potential for people living with dementia [21] and barriers to implementation [22, 23]. In general, the reviews have shown positive effects for people living with dementia and their caring unit; however, recommendations remain cautious due to the considerable heterogeneity in the nature and individual components of the roles implemented. In addition, many studies included in the systematic reviews do not adequately describe the goals of the interventions, components of the role/model implemented (including controls), methods of intervention implementation, tasks and components of the roles, and outcome measures used [25, 26]. A recent editorial of case management systematic reviews recommended that a more thorough analysis of available evidence is required in order to seek conclusions about the components of the roles that appear most successful for health, social and physical outcomes for people living with dementia and their caring unit [25].

In line with the need for more clear conclusions on what components of the roles are most successful, there is limited literature which outlines comprehensive frameworks for the implementation of community dementia key worker roles in practice. Research has been conducted on: synthesising workforce competencies and levels of practice for the dementia care workforce in general [27] and in the nursing field [28]; developing a competency framework for dementia support workers in the community [29]; and developing a skill set and job description for a dementia case manager in primary care [30]. However, the developed frameworks have primarily focused on determining what knowledge and skill areas a person working in the dementia field requires, with no great detail on: what an organisation needs to provide; what resources are needed; what the role involves if not a case management role; the personal attributes most valued; or the philosophies needed to guide the role. In addition, direct input from people living with dementia and an analysis of real-life roles currently operating in practice is missing from these frameworks.

Currently, there is also a lack of evidence on the development and implementation of key worker roles in Australia. A recent analysis by KPMG found that despite the frequent use of Australian Home and Community Care Dementia Advisory Services as funding sources for key worker type services, there is a lack of meaningful data available to assess the effectiveness of these roles [31]. One of the recommendations for maximising coverage and enhancing services in Australia included the need for a consistent dementia key worker model which provides a point of contact, during the course of the disease, for people of all ages and diverse needs groups [31].

To address the above outlined recommendations and dearth of Australian evidence, the aim of this evaluation study was to develop and describe an optimised key worker framework for people with dementia, their family and caring unit living in the Australia community.

## Methods

A number of processes were undertaken to inform the development of an optimised key worker framework including: a systematic review of the literature; an evaluation of dementia key worker models operating in Australia and an expert working and reference group.

### Systematic review of literature

International and national academic papers and grey literature focusing on key worker type roles for people living with dementia, their family and caring unit were identified by searching scientific databases, Google scholar and a variety of dementia and aged care websites. The systematic review aimed to draw out the essential components of the key worker role from high quality evidence. Criteria for inclusion included: written in the English language; published between 2003 and December 2014; the utilisation or discussion of a key worker role; participants of the study having a diagnosis of dementia, experiencing cognitive decline or being carers of people living with dementia; and research conducted in a community setting only. Once an article was deemed eligible two researchers critically appraised and assessed the articles in order to determine the quality of evidence and how well the studies were conducted in order to eliminate bias. This included how the subjects were selected, allocated to groups, managed and followed up and how the study outcomes were measured. Validated tools used to assess the quality of the articles according to study design included: Cochrane Risk of Bias Tool, Critical Appraisal Skills Programme Checklists and Greenhalgh & Taylor’s [32] qualitative framework. Full methodology and results are reported elsewhere [26].

### Evaluation of Australian key worker models

A qualitative study design was used to investigate Australian dementia key worker models operating in the community from the perspectives of three groups of participants: organisation managers; key workers; and consumers (people living with dementia and their caring unit). Ethics approval was obtained from the Royal District Nursing Service Human Research Ethics Committee (project number 149).

#### Participant selection

Purposive sampling combined with knowledge from the Expert Working and Reference Group (EWRG) was used to identify dementia key worker models currently operating in Australian community settings and managers in charge of each model. Once identified, a member of the research team (ER) contacted managers via telephone to verbally explain the research study. If organisation managers agreed to participate a snowballing approach was used whereby managers identified names of key workers currently performing in the role, and key workers identified consumers who were currently utilising or who had recently utilised the services. In some instances, consumers provided names of other people living with dementia and/or carers that they thought would be interested in participating in the study. The aim was to interview one organisation manager, one key worker and multiple consumers from each model.

#### Sample

Sixteen key worker models were identified across Australia with 14 agreeing to participate in the evaluation (Table 1). One model was run nationally while the rest were specific to one state or territory. The dementia key worker models were located in health services (*n* = 8), community care organisations (*n* = 5), a primary care setting (*n* = 1) and a consumer advocacy organisation (*n* = 1). Nine models were designed to support people living with dementia from all age groups; four (models: five; eight; twelve; fourteen) were specifically designed to support people living with younger onset dementia and one (model nine) was specifically designed to support people living with dementia aged 65 and over. The participant sample included: 37 females; 11 males; five people living with dementia and ten carers. Five of the consumers were from Victoria, five from New South Wales, two from Western Australia, two from South Australia and one from Queensland. One consumer had accessed both models three and four and therefore was able to provide feedback on their experiences with both models in the same interview.

**Table 1:** Interviews Completed

Model	State	Organisation Manager Interview	Key Worker Interview	Consumer Interview
One	Queensland	✓	✓	x
Two	New South Wales	✓	✓	✓
Three	New South Wales	✓	✓	✓
Four	New South Wales	✓	✓	✓
Five	New South Wales	✓	✓	✓
Six	New South Wales	✓	✓	x
Seven	Victoria	✓	✓	✓
Eight	Victoria	✓	x	x
Nine	Victoria	✓	x	x
Ten	South Australia	✓	✓	x
Eleven	South Australia	✓	✓	✓
Twelve	Western Australia	✓	✓	x
Thirteen	Western Australia	✓	✓	x
Fourteen	National	✓	✓ (8)	✓ (10)
TOTAL		14 interviews	19 interviews	15 interviews

*NB* For model fourteen interviews were conducted with one key worker from each state and territory in Australia. **✓** indicates same consumer

#### Data collection

Information sheets and consent forms outlining the purpose of the study were sent to all interested participants before any data was collected. Once informed consent was received, semi-structured interviews were conducted by one of two researchers both with background knowledge in dementia and no prior relationship with participants. One researcher (DG) led the organisation manager interviews while another (ER) led the key worker and consumer interviews. Both interviewers were present for the majority of interviews to ask additional questions as required.

Managerial interviews focused on organisation factors including: model design, criteria and logistics; funding arrangements; and evaluation methods. Key worker interviews were used to investigate the function of role including: logistics (mode of support, caseloads and frequency of contact); main aspects; and experience needed. Consumer interviews aimed to provide a real-life experience of how well the roles were operating in practice (see Additional file 1).

The majority of the interviews were conducted via telephone (*n* = 44); with four face-to-face interviews conducted with consumers in a home (*n* = 2) and office setting (*n* = 2) as requested. Duration of the interviews varied between 14 and 69 min.

Interviews were audio-recorded, professionally transcribed and checked for accuracy against the original recording prior to being imported into the qualitative software package QSR NVivo 10 for data management and analysis [33].

#### Data analysis

The initial descriptive topic and analytic coding was undertaken independently by one author (ER). Descriptive coding consisted of coding according to participant group and model, while topic coding was based on the interview questions. Reports combining descriptive and topic codes were analysed independently by two authors (ER; DG) using a constant comparative approach to identify themes [34]. All authors and the EWRG met to discuss and compare themes and any discrepancies or variations were discussed in workshop formats until consensus was reached.

### The expert working and reference group

This group guided the development of the optimised framework of support. The EWRG consisted of four people living with dementia, two carers of people living with dementia, 11 aged and community care industry experts, three policy experts and two researchers. All members were asked to draw on the results from the systematic review together with their expertise from research, practice or lived experience to develop an initial framework of ideal support for people living with dementia, their family and caring unit. The EWRG then used the framework to inform the interview topic guides for the different groups of participants that were consulted during the Australian evaluation (see Additional file 1). Data from the Australian evaluation was then compared and contrasted against the preliminary framework with the researchers and EWRG working together to determine essential components that formed part of the optimised framework of support for people with dementia, their family and caring unit living in the community. These processes ensured that the resulting optimised framework of support was based on best evidence and practice within the dementia care space. The development of the framework and its components was extensively discussed and incorporated multiple views. All final components were checked by the research team and the EWRG to ensure they were representative of the data and captured the main themes from all stages of the project. Throughout the course of the 2-year project, the EWRG held monthly consultations with the research team via telephone and came together in two interactive face-to-face workshops at the beginning and latter stages of the project. These monthly consultations were held to monitor study progress, discuss emerging themes from the systematic review and Australian evaluation of key worker models and provide advice on the framework development. The face-to-face meetings were used to critically discuss the framework development and to achieve consensus on the essential components of the framework of ideal support for people living with dementia, their family and caring unit.

## Results

The components of the optimised framework of support for people with dementia, their family and caring unit living in the community were incorporated under the four key themes of: overarching philosophies; organisational context; role definition; and key worker competencies. The themes within the framework are illustrated with quotes from the Australian evaluation of key worker models in Tables 2, 3, 4 and 5. Tables 2, 3, 4 and 5 also highlight which research strategy (systematic review, evaluation of Australian dementia key worker models, EWRG) the components of the framework were identified from.

**Table 2:** Essential Components - Overarching Philosophies

Overarching Philosophies	Key Components	Source	Quotes from Australian Evaluation of Dementia Key Worker Models
Relationship-centred	● Relationship- centred care should be the foundation for the key worker role	Systematic Review, EWRG, Australian Evaluation	*“I think it’s all about relationship-building... that’s how you gain trust, you build that relationship, but if you say you’re going to do something, you need to do it”* [Key Worker Seventeen] *“Once you have someone walking alongside you like that asI said it can be the difference between a carer actually crumbling in their health.”* [Consumer Seven]
Enablement	● Retaining a sense of self and identity ● Focusing on strengths and building resilience ● Planning for the future to provide peace of mind ● Supporting the person to continue to live their life as they choose ● Protecting the rights of people to full citizenship regardless of their age, gender, cultural background	EWRG, Australian Evaluation	*“I’m looking at who they are as a person. I’m interested in their history, so that we can actually support that and validating who they’ve been, what their likes are what their dislikes are.”* [Key Worker Five] *“I think there’s a degree of give and take in this. While I’m well enough to manage, then I will give and say no, I can manage this... her role is to guide me how to intervene for myself.”* [Consumer Twelve]
Holistic	● Encompassing emotional, social, physical and spiritual dimensions of support ● Providing a whole of family/caring unit response to support ● Recognising support will need to be more frequent and intense at different times with different people ● Recognising the relationship that remains between the person with dementia and their caring unit ● Inter-professional and inter-sectoral collaboration	EWRG, Australian Evaluation	*“We take a pretty holistic approach to the whole situation. The person who is living with dementia, it’s very, very hard to separate that person out and provide them with support and not look at the family system that they live with.”* [Key Worker Eighteen] *“I think it’s not just the physical help that you need, it is this emotional and supportive role that is so important. It’s definitely got us to where we are now. I truly believe that without the help from [key worker], I think that the only decision open to us would have been to put my uncle into permanent care.”* [Consumer One]
Accessibility	● Diversity, access and equity awareness (rural, regional areas; diverse groups) ● Having access to support where there is diminished decision making capacity ● Retaining respect and advocating for the rights of all people ● Having access to palliation and end of life care in the setting of advanced or impaired decision making ● Working with the person and those who support them regardless of age	EWRG, Australian Evaluation	*“Adaptable, it’s flexible, it covers everything throughout the continuum of the journey... it’s one place for people to actually be given a clue as to what you might need in the future. Giving people what their options are and what times they should be looking into the next option before they need it. Somewhere they know that they’ll be respected and whoever is in this position will try their best to make their lives a little bit easier. An inclusive, holistic person centred approach.”* [Key Worker Four] *“I’m really hopeful that I will be able to keep having [key worker] presence in my life, because I would say to you now, without her presence in my life - even as a case manager whose primary role at the moment is to act as an advocate and as to be a support person to me, to provide that emotional and psychological support that I need - without that...I would be overwhelmingly isolated, as somebody with younger onset dementia, living in the community on my own.”* [Consumer Twelve]

**Table 3:** Essential Component: Organisational Context

Organisational Context	Key Components	Source	Quotes from Australian Evaluation of Dementia Key Worker Models
Model Design	● Clear model design with desired outcomes documented ● Continual support; not time-limited ● Support across the continuum ● Flexible entry and exit criteria ● Encouragement and promotion of early intervention ● Continuity of care ● Flexible mode of support and frequency of contact ● Multi-disciplinary dementia support teams with a range of professions	Systematic Review, EWRG, Australian Evaluation	*“For me I see it as an ongoing thing if something arises we would certainly have them as a client again we can absolutely justify it as we know working with people with dementia can be a long term thing.”* [Key Worker One] “[Key worker] *was available 24/7 basically. We felt sort of really comforted by the fact that we had that security... dementia is 24/7.”* [Consumer Nine] *“You need to do things early and think about all the things ahead of time and I’m so glad that we did make that connection really before we desperately needed it. Because if you reach that desperation point and you’re highly anxious and stressed, then it’s harder to make that connection. I don’t know how people would be able to manage that situation if they left it until it was the crisis point.”* [Consumer Four] *“In an ideal world you would have a multidisciplinary team because then it would be a really quality service: social worker, occupational therapist, psychologist, registered nurse*” [Organisation Manager Three]
Quality Assurance	● Formal evaluation ● Consumer feedback: follow-up with all people receiving and withdrawing from the service ● Regular auditing	EWRG, Australian Evaluation	“[Evaluation] *that is what we need to do. What we can provide is what we have in terms of feedback from carers, the referrals, assessments, case management of these patients that is all we have at this stage but I think it does need a more thorough evaluation to justify the funding.”* [Organisation Manager Two]
Inter-professional and Inter-Sectoral Collaboration	● Strong links with other dementia, services and organisations ● Integrated approach to care; not working in silos or isolation ● Investments in a strong provider network	Systematic Review, EWRG, Australian Evaluation	*“The aspect is to have that good capacity for interagency communication and building working relationships with other organisations that are relevant to the client group.”* [Organisation Manager Twelve] *“We’ve got our key result areas that are in- service sector collaboration and capacity building. An example of that would be, [key worker] and I went and did a two-and-a-half-hour training session at a disability service provider, because they had a person with younger onset dementia... they brought us in to help them build capacity amongst their staff”* [Key Worker Eighteen]
Infrastructure Support	● Support for staff well-being, skill development and performance ● Monitoring caseloads and workloads closely and frequently	Australian Evaluation	*“I definitely, in the past, have not been burnt out but been close to it. Just due to the high number of clients that I do have. That’s 53 clients but that’s also their carer and their families as well. So you’re dealing with 53 families that have such complex relationships as well, it’s easy for a worker to burn out.”* [Key Worker Fourteen] *“I mean I just don’t know how [key worker] juggles what they have to do in the time...I just think that when there’s so few it’s really hard to know how much you can expect from them because it’s unrealistic.”* [Consumer Two]
Resources	● Physical resources: maps of services; vehicles (pool cars, reimbursement for travel); computers; phones; electronic databases ● Managerial support ● Professional development (case conferencing, mentoring, education, training, conferences, ongoing study opportunities, shadowing opportunities) ● Clinical support ● Access to technology (Skype; video conferencing)	Australian Evaluation	*“They have to have a case conferencing system; but you really need a multidisciplinary team.”* [Organisation Manager One] *“Each team gets a consultation with the neuropsychology unit...we tease out the specifics of it, so we can come up with some strategies or make sure that what we’re doing we’re on the right track... and get somebody who’s external to us, look at us, tell us how are we doing here, and what do you know that we don’t that we might be able to work with.”* [Key Worker Eighteen]

**Table 4:** Essential Component: Role Definition

Key Worker Role	Key Components	Source	Quotes from Australian Evaluation of Dementia Key Worker Models
Referral and Linkage to Services	● Building relationships, linking with and collaborating with other services ● Referring to other services and professionals as appropriate ● Enabling the person to assist with referral processes	EWRG, Australian Evaluation	*“It can be putting them in touch and referring them onto services, whether that is respite, day respite, packages, homecare. I help ascertain what their needs might be and point them in the right direction I can make the referrals myself whichever’s appropriate.”* [Key Worker Four] *“The approach was primarily to facilitate the provision of services that we were looking to have... She took care of getting me a mental health plan and getting that working. She was very useful as a coordinator in the services.”* [Consumer Eleven]
Navigating the Service System	● Connecting and coordinating with appropriate services in partnership with the person being supported ● Ensuring continuity	EWRG, Australian Evaluation	*“The first area is supporting to navigate and negotiate the service maze. So to really not just know what’s available and what they can access but actually how to describe their situation, how they can be creative around those services and support so that they really work for their situation and what they need most”* [Organisation Manager Eleven] *“Well for me it was wonderful that [key worker] was the one to ring the Cottage to see if there was room for a day respite and she got that person to ring me to say, these days are available or these times, when would you like to come in and have a look at the place. So she arranged all of that, she did all of that phone work”* [Consumer Two]
Responding to Individual Needs	● Holistic discussion and assessment of physical, emotional, social needs ● Determining what is important to each person and working with them to achieve their goals ● Building relationships with the person being supported ● Utilising an enabling approach: promoting and facilitating choice, independence, wellbeing and quality of life ● Working in partnership with all parties to refine care process ● Providing practical, emotional, spiritual and social support	Systematic Review, EWRG, Australian Evaluation	“W*e talk about what sort of things a person is doing so what sort of work or interests they’ve had in their lives and that helps to guide me to where I might be placing them or helping them out with things”* [Key Worker Ten] *“Over time we’ve probably met each and every one of the workers, and there were a couple there that just weren’t really dementia people, or they just concentrated on the carers and the dementia person was just a side event. Whereas the ones we’ve got now really do concentrate on the dementia person and the carer and gives feedback and support.”* [Consumer Five]
Education and Information Provision	● Providing timely, informed and current knowledge about dementia and services Providing appropriate resources ● Mentoring role, educating and raising awareness to the broader community	Systematic Review, EWRG, Australian Evaluation	*“Sometimes it’s a bit of education about the disease, often it’s about communication and behavioural issues, giving them strategies and tips and hints language to use, body language.”* [Key Worker Four] *“A big part of what we do too is education and delivering training to communities... raise awareness and de-stigmatise dementia in the community too as well even more so in those rural areas.”* [Key Worker One] “*This is what they reinforce all the time: you don’t look at what they can’t do; you’re looking at what they can do. So you’re focusing on the positives rather than the negatives. But if you don’t have that support, I imagine it would be very easy to focus on the negative. So from a mental and emotional point of view, having that support and that education, it helps tremendously. It just gives you that bit more strength.”* [Consumer Nine]
Listening	● Listening and providing support, comfort and options/ suggestions	EWRG, Australian Evaluation	“A *lot of the time is, they just want to blurt it out and then they’re right. They don’t need any more, because they don’t want to share it with their family, because they don’t want their family to think they’re not coping.”* [Key Worker Five] *“They need to be a good listener and they need to be able to almost read between the lines. If you phone up and say [name] is bored and I don’t know what to do with him, instead of saying well, have a cup of tea, it’s a matter of okay what has he been doing and what can we do.”* [Consumer Five]
Emotional Support	● Referring to counselling services if needed ● Connecting those living alone to communities	EWRG, Australian Evaluation	“*We do a lot of counselling and therapy for the individuals, their families and their carer system and then we also link with appropriate resources”* [Key Worker Twelve] *“As the journey progresses and your partner just is deteriorating, falling apart before your eyes really, that emotional support is really important... So I just had a session with her and that was more really a counselling session that went for an hour and a half and it was terrific. I really needed it at that stage to get back on track”* [Consumer Four]
Practical Support	● Ongoing monitoring of outcomes ● Assist with problem solving skills, communication techniques and strategies for person with dementia ● Clarification of risk factors and parameters set by each person ● Rehabilitation: enabling people to achieve their optimum physical, emotional, spiritual and social wellbeing	EWRG, Australian Evaluation	*“Recently they’ve come up with - and made personally - a much more practical daily diary for [person with dementia]. I just Velcro what we’re doing that day onto it instead of having to write it out. She’s waiting for photographs of what’s inside our cupboard so she can print and Velcro photos to help in the kitchen a bit more.”* [Consumer Five]
Point of Contact	● Being a central point of contact ● Continual support as required Recognition of when to step in and out triggered by the person identifying a need for support and proactive follow-up	Systematic Review, EWRG, Australian Evaluation	*“We try and encourage a sense of this is a resource or centre that you can always ring back and link with if any difficulties occur and it does seem to work...they don’t have to run around to a hundred different places this is the kind of the centre where the support is localised.”* [Organisation Manager Twelve] *“For me it’s the underlying security. A key worker is almost like a safety net. If there are any problems, then that’s my first port of call. So far they’ve helped I think in every situation I’ve taken to them. I can’t think of one where it hasn’t”* [Consumer Five]
Advocacy	● Individual advocacy; seeking solution with and for people with dementia to their particular problems or needs ● Linking with and advocating for appropriate services and ensuring that referrals are followed through	EWRG, Australian Evaluation	*“So I am able to empower her and her family in what is dementia so that when they go to a GP they can actually have more of an accurate conversation with them about the changes that they have noticed... we are just there to have a chat to and say you know make sure you talk about this aspect of her behaviour changes... I guess that advocacy is also part of our skill set as well”* [Key Worker Nineteen] *“If I need to go to my accountant and I need a second set of ears, to listen to what’s being said, she will come with me. Where I request her to come to something, she will come along and just be there...it’s just a reminder to the other party, hey, there’s somebody interested in what’s going on here. So just tread carefully. The fact that you say, I’m here as her advocate if she needs something - just silently alerts them to the fact that there is a responsibility that they have towards my wellbeing and interests.”* [Consumer Twelve]

**Table 5:** Essential Components: Key Worker Competencies

Key Worker	Areas	Source	Quotes from Australian Evaluation of Dementia Key Worker Models
Knowledge	Consensus Areas: ● Dementia Other Areas: ● Clinical and Scientific (e.g. nursing background, allied health background) *(10 models)* ● Family and Carer Needs (e.g. utilising a whole of family approach to support) *(eight models)* ● Services and Resources (e.g. service availability, referral pathways, entry criteria, waitlists, education/training opportunities) *(eight models)* ● Sector (e.g. aged care, disability) *(six models)* ● Work Experience *(five models)* ● Life Experience *(three models)* ● Cultural Differences *(two models)*	Australian Evaluation	*“You’ve got to have a spread of knowledge. I think the support worker needs to be embedded in a dementia specific knowledge base.”* [Key Worker Sixteen] *“I believe that they need to have an understanding of or some type of training in aged care, especially with people with dementia. They need to really get to know that person”* [Consumer Eight]
Skills	Consensus Areas: ● Communication and interpersonal ● Ability to generate ideas and problem solve ● Ability to build and maintain relationships Other Areas: ● Ability to provide education (information provision) *(11 models)* ● Clinical (e.g. ability to judge and screen for medical issues, medication issues, physical changes) *(six models)* ● Counselling *(five models)* ● Advocacy *(five models)* ● Assessment (e.g. ability to screen and assess physical, social, mental, emotional health needs) *(four models)* ● Time Management *(four models)*	Australian Evaluation	*“Definitely the people skills and being able to ask the questions that a lot of people wouldn’t normally consider. It’s not just as simple as asking a question. You actually can then offer some solutions for those questions that they have. You have to be I guess quite good at communicating with people in a variety of different contexts whether that’s phone or email or face to face.”* [Key Worker Twenty] *“I mean it’s like going to a restaurant, you get given a menu. There’s no point saying well I want sort of scrambled deer antlers and they say, oh sorry we don’t have any and you’ll have to get something else... tell us what you do have and then we can go from there because most people aren’t going to want everything. They’re just going to say, well this would be helpful. I don’t want that or that or that but I wouldn’t mind that and perhaps down the track I’d like to have a bit of that. So just some suggestions so that you know what is on offer, what is available.”* [Consumer Two]
Attributes	Consensus Areas –Key Workers, Organisation Managers *(top five):* 1. Empathetic *(ten models)* 2. Good Listener *(seven models)* 3. Person-centred *(six models)* 4. Adaptable and Flexible *(five models)* 5. Genuine, Kind, Caring *(five models)* Consensus Areas – Consumers *(top five)* 1. Good Listener *(six consumers)* 2. Empathetic *(four consumers)* 3. Caring *(three consumers)* 3a) Stable, Reliable, Committed *(three consumers)* 3b) Proactive *(three consumers)* 3c) Perceptive, Astute *(three consumers)* 3d) Practical, Realistic *(three consumers)* 4. Understanding *(two consumers)* 4a) Resilient *(two consumers)* 4b) Open *(two consumers)* 5. Encouraging *(one consumer)*	Australian Evaluation	*“Empathy is really, really important, but also the ability to hold their hand and go, okay, I’m here, I’m listening to you. There are things we can do together and then let’s see how we can build your strength to be able to cope with this.”* [Key Worker Five] “*Just being able to listen I think is a key thing... it’s the first time they have been able to talk to someone that doesn’t have an emotional investment in making some decision or judging them*.” [Key Worker Ten] *“They need to be empathetic, understanding, be prepared to listen, be practical. You know, there’s none of that airy fairy stuff. You really have to be practical and realistic about it”* [Consumer Nine] “*They need to be a good listener and they need to be able to almost read between the lines.”* [Consumer Five] “*I think the biggest thing for me was just having someone to sound off to that was actually not just sympathetic, empathetic. She was actually encouraging and she’d give me ideas of how she handled things. She encouraged me to be brave”* [Consumer Three]

### Overarching philosophies

The overarching philosophy component of the framework incorporated a number of more clearly defined subthemes of support including: relationship-centred; enablement; holistic; and accessibility. These themes are described below and classified in Table 2.

#### Relationship-Centred

The capacity to build and maintain relationships with people living with dementia, families, the caring unit, health professionals, service providers and the sector in general was reported to be central to the key worker role. This was the strongest theme coming out of the EWRG and the Australian evaluation and also evident in the systematic review. Organisation managers and key workers stated that this ability helped put people at ease, validated their emotions and promoted trust and safety. Building relationships with services and health professionals was reported to lead to better quality of care for the people the workers supported and assist with the transfer of information, resulting in less duplication amongst services.

#### Enablement

The need for the key workers to use an enabling approach came out strongly through the EWRG and the Australian evaluation. In particular, people living with dementia wanted key workers to focus on their strengths not deficits, self-identities, maintenance of their pre-diagnosis way of living and their right to live their life the way they choose. Carers and people living with dementia highly valued key workers who promoted a collaborative approach to the care and support provided and actively involved them in any goal setting and/or action plans – themes that were also strongly evident in the systematic review of literature.

#### Holistic

Holistic support that incorporated emotional, social, physical and spiritual dimensions was highly valued by all respondents in the Australian evaluation and viewed as essential by the EWRG. This included ensuring support met individual situations and issues and the key workers gained background knowledge of the person and their history. This holistic aspect of support was also linked to the two previous philosophies of promoting enablement and building trusting relationships with the person and all parties involved in the care process.

#### Accessibility

The EWRG and respondents in the Australian evaluation reported that in an ideal world a key worker model would be accessible to people of all ages, diverse needs groups, across all stages of dementia (pre-diagnosis, post-diagnosis, early, moderate, advanced dementia) and the continuum of care (primary, community, acute, residential aged care sectors). Effective planning and decision making to support immediate needs and plan for future preferences was also viewed as essential. Consumers highly valued early intervention and continual support with the flexibility to contact the key worker at any point as needs changed. It was particularly noted how vital it was to have immediate access to a key worker to make connections before people became stressed, anxious or were potentially at crisis point.

### Organisational context

Through each process of the framework development organisational factors for implementing a dementia key worker model were raised. These factors were broadly categorised into: model design; quality assurance; inter-professional and inter-sectoral collaborations; infra-structure support; and resources. These aspects are described below and defined in Table 3.

#### Model design

Important aspects of model design identified through the ERWG, Australian evaluation and systematic review included: flexible entry and exit criteria; continual support that is not time limited; support across the continuum; continuity of care (limiting staff turnover); flexible mode of support and frequency of contact; early intervention and multi-disciplinary teams. In particular, multi-disciplinary teams were thought to lead to high quality, holistic models of support.

#### Quality assurance

The EWRG viewed regular auditing and formal evaluation measures as essential components of ensuring a quality service. In the Australian evaluation, organisation managers reported that without quality assurance and formal evaluations it was hard for them to quantify the benefits or cost-saving capabilities of their models or determine why people withdrew or didn’t engage with their services. The majority of respondents felt it was particularly important to gain views on the service directly from people living with dementia and their caring unit in order to gauge how well the service was working in practice.

#### Inter-professional and inter-Sectoral collaboration

Through the EWRG, Australian evaluation and systematic review the importance of inter-professional and inter-sectoral collaboration was strongly apparent. Respondents particularly commented on the importance of integrated approaches to care and the need for organisations to not work in silos or isolation. Further, evidence from the systematic review showed that investments in strong provider networks including linking with and having close contact with the physicians/General Practitioners of each person was important for an ideal model of support.

#### Infrastructure support

Regular reviews of work and case-loads as well as formal self-care processes were stated to be vital aspects of organisational support. Generally key workers in the Australian evaluation felt they had support within their own team but there were limited formal processes set up for them to access de-briefing and counselling services. High caseloads also impacted on the key worker’s ability to perform the role effectively and on consumers who often chose not to contact their key worker for fear of taking their attention away from other people who “*needed their support more*”. Being burnt-out, fatigued and personally affected by situations were feelings reported by several key workers.

#### Resources

Through the Australian evaluation, a number of resources needed to support the implementation of the role were raised including: environmental map of services; computers; vehicles; phones; electronic databases; managerial support; professional development; clinical support; and access to technology. Case conferencing in particular was reported to be vital for ongoing professional growth and highly valued by key workers as it gave them an opportunity to discuss issues, determine strategies and gain a broader understanding of different approaches.

### Role definition

**Fig. 1:**
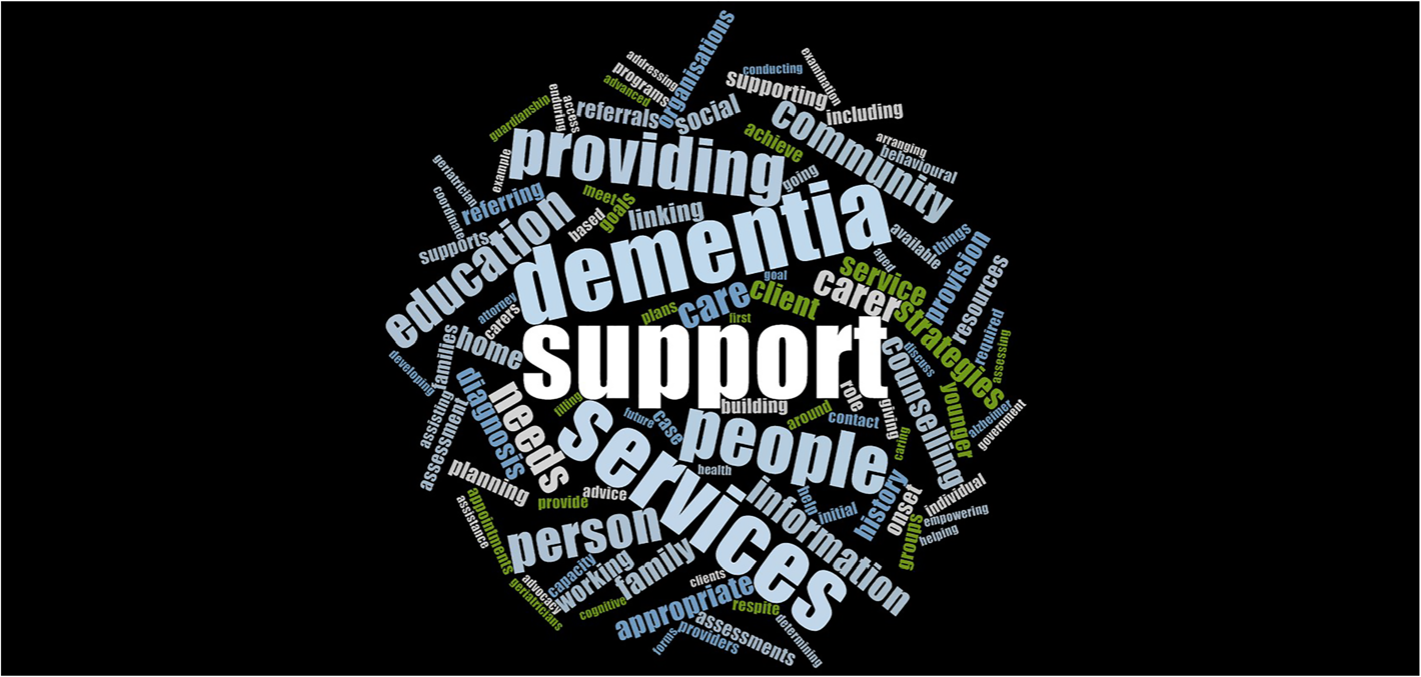
Role of a Key Worker Word Cloud

The key worker role was very broad. Respondents from each of the 14 models identified over 152 different tasks the key workers undertook in their role (Fig. 1). The key finding was that the role was very diverse and changeable; there was no day that was the same or person that required the same support.

*“We are dealing with a broad range of issues they are not all just dementia related issues ... there are a lot of social issues, personal issues, or relation-based issues.”* [Key Worker One]

*“Every day is very different and every client as well and every carer - their situations are all very unique... we do a lot.”* [Key Worker Fourteen]

Content analysis revealed that the most common aspects relating to the key worker role were: support (32 times), services (26), education (14), needs (12), care (10), information (10), counselling (8), strategies (8), diagnosis (7), history (6), linking (6), provision (6), services (6), planning (5), referrals (5), resources (5), assessment (4) and goals (4).

Despite the diversity and changeability of the role, there were common components across the models evaluated. The main role aspects reported by organisation managers and key workers included: referral to services (14 models); navigating the service system (14 models); responding to individual needs (12 models); providing education and information to people living with dementia/caring unit (10 models); listening (9 models); point of contact (9 models); and providing education and training to the community (5 models). In addition, consumers reported 55 ways that their key worker had been able to assist them. These support methods generally fell into five categories: providing information, education and knowledge; referral and linkage into services; practical support; emotional support and advocacy. These aspects of the role have been collated and clearly defined in Table 4.

### Key worker competencies

A major theme arising from the Australian evaluation of dementia key worker models was the need to get the right person in the key worker role. Organisation managers and consumers reported that some key workers had not suited the role and this was thought to come down to their personal attributes or a lack of experience (professional and personal), commitment and passion for the role.

*“I've had people who have been beautifully qualified but they haven't got an emphatic bone in their body, the clients know.”* [Organisation Manager Four]

*“Everything I said was valid. It wasn't put in the too hard box sort of thing, you know. I was quite disappointed with the other two services...I thought well they obviously didn't listen but this [key worker] really was perceptive... she was a really good listener too”* [Consumer Three]

The knowledge, skills and attributes thought to be essential for the role are described further below and in Table 5.

#### Knowledge

Eight knowledge areas thought to be essential to the role were raised in the Australian evaluation including: dementia, clinical and scientific, family and carer needs, services and resources, sector, work and life experience and cultural differences. Knowledge of dementia was the only area validated by all respondents and models. While dementia specific knowledge was seen as a vital component of the role, the majority of respondents in the Australian evaluation felt that you did not need to come into the role with a pre-existing dementia qualification and that the ability to learn and receive training on the job should be an essential component in all key worker roles. In addition, personal attributes, the ability to learn and change, work and life experience and willingness to seek information were seen as more important than a certain professional background. All respondents also acknowledged that while there were a variety of professional backgrounds that made someone suitable for the role it was essential to have a spread of knowledge ideally in the form of a multi-disciplinary team.

#### Skills

It was felt by all respondents that the key workers needed a broad range of skills to perform their role effectively. Overall, 12 skill areas were raised: communication and interpersonal skills; ability to generate creative ideas; ability to build relationships and network; ability to provide education and information; ability to judge and screen for clinical issues; counselling; advocacy; assessment and time management. Only three skill areas (communication and interpersonal skills; the ability to generate creative ideas; and the ability to build relationships and network) were reported by all respondents and models. Consumers in particular valued a worker’s ability to offer up creative and innovative solutions to any problems and issues discussed. This skill was also referred to as being the “*ideas person*” and “*advise*r”.

#### Attributes

When discussing knowledge, skills and attributes needed for the key worker role it was often hard for respondents to distinguish between what constituted a skill and what constituted an attribute. Despite this personal attributes of the key worker were commonly reported to be more important than knowledge or skill areas that could be taught on the job. Over 50 attributes were raised by respondents in the Australian evaluation with empathy and being a good listener being reported the most.

## Discussion

**Fig. 2:**
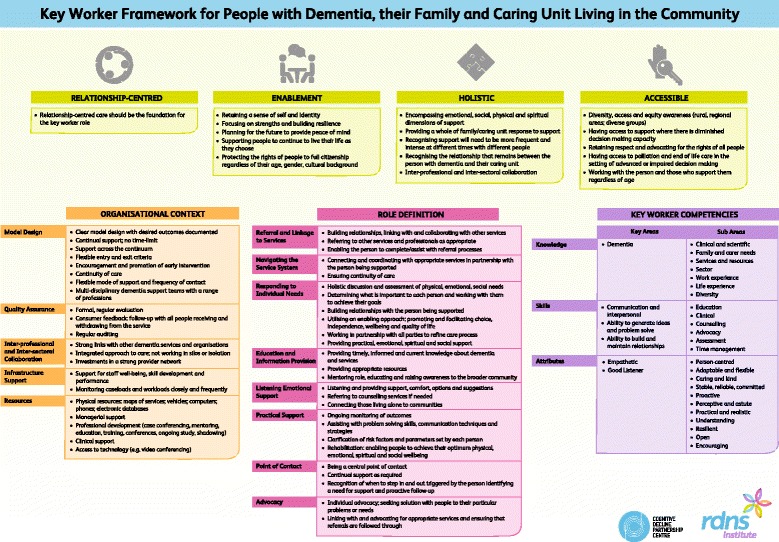
Optimised Key Worker Framework for people with dementia, their family and caring unit living in the community

In this paper an optimised key worker framework for people living with dementia, their family and caring unit has been developed and presented (Fig. 2). The framework draws on an extensive evaluation of literature, numerous Australian dementia key worker models and direct feedback from practice. This multi-method approach resulted in the establishment of an evidence-base for all components of the key worker framework. The optimised framework has been designed and validated by numerous stakeholders including: people living with dementia and their caring unit; key workers; managers; industry experts; researchers; and policy makers. Often consumer perspectives are not taken into account in the development of frameworks or models; this framework has been driven and designed by consumers at all stages of development from inception to completion. In particular people living with dementia and carers provided key voices in the design highlighting direct experiences of current key worker models as well as true insight into what it is like to live with dementia every day.

The evidence-based framework reflects the complexity of the dementia journey and the difficulty of trying to develop a ‘one size fits all’ approach. It highlights the reality of life for people living with dementia and the challenges policy makers, funders and service providers face in order to address the complexities of the disease and the journey of those involved. It outlines key philosophy areas and important organisational considerations that should be used to guide the implementation of current and future key worker models. Additionally, it outlines clear role descriptions as well as the key competencies and specifications of an ideal dementia key worker.

In line with the previous competency frameworks developed for dementia care this framework outlined and validated a number of key skill and knowledge areas thought to be vital for the key worker to possess. However, a key point of difference in this optimised framework was the placement of personal attributes as substantially more important than skills or knowledge that could be taught on the job. In particular, people living with dementia and their caring unit highly valued personal attributes noting that workers without the right qualities were harder to form trusting relationships with. The two attribute areas reported to be essential to the role were empathy and being a good listener. Empathy is the experience of understanding another’s condition from their perspective while being a good listener can also help place yourself in someone else’s position, enrich your understanding of needs and expand your capacity for empathy. Given these findings it should be considered essential to focus on personal attributes when recruiting for dementia key worker roles. Therefore, personal attributes separated from knowledge and skill areas form a key component of this developed framework.

This evaluation also drew out four philosophical areas that were viewed as vital components of the role (relationship-centre care; enablement; holistic; and accessibility). In particular, building and maintaining relationships was reported to be central to the key worker’s role and was the strongest theme coming out of all the consultations conducted as part of the evaluation. Increasingly relationships are being viewed as critical to the care provided by all practitioners, a source of positive outcomes, and a prerequisite to effective care. The phrase relationship-centred care was viewed as capturing the importance of the interaction among people as the foundation of any therapeutic or healing activity [35]. Previous frameworks by Waugh and colleagues [30] and Tsaroucha and colleagues [27] also point to the importance of incorporating, within worker’s skill sets, the ability to establish and maintain trustful and respectful working relationships between all people involved in their care and support. This framework goes one step further, pulling relationship-centred care out of just being part of a skill set and placing it as a vital overarching philosophy area that guides the entire key worker framework of support.

One of the key findings through this evaluation was how diverse, broad and changeable the key worker role is. However, despite the broadness the role was able to be summarised into nine categories of support with clear descriptions under each category. This categorisation, which provides a clear role description, will prove helpful for future research studies wishing to implement and evaluate key worker type roles. Implementation of this key worker framework will also help address previous concerns that past studies have not sufficiently described what the key worker roles involved or the components of the role or model. This will allow future systematic reviews to attribute successful and non-successful outcomes and observations directly to the key worker role and if implemented and described appropriately to distinct components of the key worker role interventions.

Furthermore, through the development of this framework organisational factors and resources needed to support the workers in their role were identified which were not drawn out in the previous competency frameworks. Overall, the main organisational context components included: clear model designs; multi-disciplinary teams; quality assurance; evaluation; consumer feedback; inter-professional and inter-sectoral collaboration; infrastructure support and resources. A recent systematic review found that in particular optimal caseloads, target populations (person-centred approaches), delineation of the case managers role and their characteristics (interdisciplinary care protocols) facilitated successful case management implementation in primary health care [23]. A further systematic review by the same authors showed that the components of ‘high-intensity’ case management were key factors in optimising service use and producing positive clinical outcomes for the person with dementia/caring units [22]. These components included: small caseloads; regular meetings with people living with dementia and caring units; education on health conditions; close contact with family physicians; and proactive follow-up. These systematic review findings match and add further validation to this framework’s essential components of support [22].

### Practice and policy implications

This evaluation showed that currently there is little consistency in Australian dementia key worker models. Furthermore, there is not one model that is accessible to people of all ages, diverse needs groups or geographic locations. The Australian key worker models used to inform the developed framework were located in a variety of organisations and operated under different organisational contexts and funding structures.

There is great opportunity to use the developed framework to guide the implementation of a dementia key worker model accessible nation-wide. One of the key principles of the framework is individualised support meaning that regardless of one’s background or diversity the support provided should meet their needs. Implementation of a key worker role guided by this evidence-based framework has the potential to provide optimised, high quality support to all people impacted by dementia. Situating the dementia key worker role outside of one single organisation would also allow a consistent model of support that is accessible for all people living with dementia, their family and caring unit and enable greater inter-professional and inter-sectoral collaboration.

One of the key recommendations from the newly published Clinical Practice Guidelines for Dementia in Australia is to ensure people living with dementia have access to a care coordinator/ key worker who works with them and their family and caring unit from the time of diagnosis [36, 37]. Further, it is recommended that services should agree on one main contact point/service who is responsible for coordinating care across services; points that are all validated by this research [36, 37].

This developed framework also has the potential to be utilised as a tool for current dementia key worker services in regards to human resources recruitment and service review and development. It could also be used to assess the competencies of current dementia key workers and develop specific dementia training and education courses to enhance professional development. One of the main findings through this process was the breadth of current key worker roles. The role was reported to be all encompassing, broad and varied which impacted on the workers (physically and emotionally) and subsequently the people being supported. Utilisation of this evidence-based framework with its clear requirements for organisational support, work conditions, job descriptions and key knowledge, skill and attribute areas has the potential to support current workers in a more comprehensive way and as such improve quality of care and experiences for people living with dementia, their family and caring unit.

### Next steps

Despite the framework’s potential for use in the Australian context further evaluation of its effectiveness and relevance for practice within the dementia care space is required. Such evaluations should be high quality, robust and take place across several Australian states to ensure the framework is appropriate to the needs of people living with dementia, their family and caring unit in a variety of settings. Active involvement of service providers, people living with dementia, caring units and policy makers will be essential for successful evaluation and implementation of the framework.

### Limitations

The developed framework is preliminary and requires further testing. The main limitation related to the inability to gain consumer or key worker views on all of the models identified in the Australian evaluation. The reasons for interview refusals varied but primarily could be attributed to accessing contacts through gatekeeper organisations. The self-selection of some of the participants also means the findings cannot be generalised to all key workers, people living with dementia and caring units. However, the majority of participants talked openly about the key worker services, raising crucial strengths and limitations that were used to inform the optimised framework.

In addition, despite the utilisation of a comprehensive search strategy it is possible that some dementia key worker programs in operation in Australia and international or national dementia key worker literature were not identified. This review also focused solely on interventions that were community-based and didn’t investigate support models for people living with dementia, their family and caring units that were purely based in residential care or hospital settings.

## Conclusion

An evidence-based key worker framework has been developed for people with dementia, their family and caring unit living in the community. The framework outlines a comprehensive synthesis of components viewed as being essential to the implementation of a dementia key worker model of care. Its strength and novelty lies in its multi-method, co-designed approach to development. The framework has great applicability to dementia care services in Australia and has the potential to assist organisations, service providers, governments and policy makers to provide high quality care and consistent support to all people living with dementia, their family and caring unit.

## Additional file

Additional file 1: The interview topic guides used for each group of participants that took part in the evaluation of Australian Key Worker Models (organisation managers; key workers; people living with dementia; caring unit/families) are in included as a supplementary file. (DOCX 15 kb)

## Abbreviations

EWRG: Expert Working and Reference Group
