# Supplementary material for: Development of an optimised key worker framework for people with dementia, their family and caring unit living in the community
Source: BMC Health Serv Res. 2017 Jul 20;17:501. doi: 10.1186/s12913-017-2448-0 (PMC5520382; doi:10.1186/s12913-017-2448-0)
Supplement: Additional file 1: — The interview topic guides used for each group of participants that took part in the evaluation of Australian Key Worker Models (organisation managers; key workers; people living with dementia; caring unit/families) are in included as a supplementary file. (DOCX 15 kb) [file 12913_2017_2448_MOESM1_ESM.docx]

**Interview Topic Guides**

# Organisation Managers

1. Can you tell me about your organisational context?
2. What resources are required to support a key worker?
3. How does the position fit within your organisation?
4. Do key workers accept referrals internally to the organisation or from external referral points?
5. What are the quality improvement processes?
6. Does the position fit within a Consumer Directed Care Model?
7. Does the key worker have the ability to adapt to meet the needs of those living with younger onset dementia?
8. Did you conduct an environmental plan/mapping of available services as a resource for use by key workers? Or do the key workers do this?
9. How many people receiving key worker support do you have on your books?
10. Do key workers have a formal entry and exit criteria?
11. Is there flexibility in the key worker program to build working relationships with clients and families?
12. Is there potential to replace the key worker if they are unable to establish a positive relationship with the client?
13. How do you monitor service activity with the program?
14. Do you have a quality process supporting client service satisfaction?
15. How many people pull out of the service? What reasons do they give for pulling out of the service?
16. Why have you chosen to support a key worker dementia model of care in your organisation/s?
17. How can the role best be utilised to assist people living with dementia and their carers living in the community?

Funding and cost-effectiveness

1. What is the cost of providing this role?
2. How is the position funded?
3. Is funding ongoing? At risk?
4. Have you evaluated the key worker role for cost effectiveness?
5. What about evaluating other measurers?
6. How many people can one key worker see per week?
7. How many cases can a key worker manage?

Benefits

1. What is the benefit of providing this role?
2. Does the key worker service lead to improved access to services to maintain independent living?
3. Is there a reduction in admissions to residential aged care or hospital?
4. Is there an improvement in co-ordination of services?
5. Is there an improved ability to manage at home?

# Key Workers

1. What is your understanding of the key worker role and what it involves?
2. What does a key worker do for clients?
3. What skills would you say a key worker requires?
4. What is the normal case load of a key worker?
5. Does the inclusion criteria for a key worker depend on a diagnosis of dementia?
6. On average how long has someone had a diagnosis of dementia prior to your making contact with them?
7. Are there any wait lists for key worker services?
8. Are there any people you don’t accept? Why not?
9. Is there a cost for the service?
10. Does the position service rural/regional areas?
11. Does the service vary depending on location eg rural/regional?
12. How could the model be adjusted for people of all ages (younger and older onset dementia)?
13. What proportion of your clients are culturally and linguistically diverse?
14. How frequently do you visit your clients?
15. How frequently do you contact your clients?
16. What organisational constraints are there that impact on your role?

Outcomes and effectiveness of the role

1. Has there been any evaluation of the role?
2. Were you satisfied with the outcomes achieved?
3. Did consumers/carers find the role effective?
4. Is there potential to replace the keyworker if they are unable to establish a positive relationship with the client?
5. How many people pull out of the service? What reasons do they give for pulling out of the service?
6. How can the role best be utilised to assist people living with dementia and their carers living in the community?
7. What are the limitations/strengths of the service? What do people benefit from most?

# People living with dementia

1. Tell me about your experience of living with dementia?
2. What do you think a key worker does? What does their role cover? What should they do?
3. Have you used/experienced a key worker service?
4. If so please describe how you found out about or came in contact with the key worker service?
5. Was it easy to be referred to the service, could you self refer?
6. Were you provided any information about the service? If so was it useful?
7. What was your experience of the key worker service?
8. What contact did you have with a key worker?
9. Were you satisfied with their knowledge about dementia, ability to find services?
10. What were the outcomes of having a key worker for you?
11. Were you satisfied with the outcomes achieved?
12. How long did you know you had dementia before you accessed the services of a key worker?
13. Did you feel you could contact the key worker outside of scheduled meeting times if something came up?
14. Would you be receptive to using the internet or telephone to communicate with your keyworker?
15. Did you have a choice of key worker?
16. What skills does a key worker require?
17. How can the role best be utilised for people living with dementia/carers such as yourself?
18. Were there any gaps in the service?
19. What are the benefits of having a key worker?
20. Are there any questions we have not asked that you think we should be asking?

# Caring Unit/Families

1. Tell me about your experience of caring for someone living with dementia?
2. What do you think a key worker does? What does their role cover? What should they do?
3. What was your experience of the key worker service?
4. Were you satisfied with their knowledge about dementia, ability to find services?
5. What were the outcomes of having a key worker for you?
6. Were you satisfied with the outcomes achieved?
7. How long did you know the person you care for had dementia before you accessed the services of a key worker?
8. Did you feel you could contact the key worker outside of scheduled meeting times if something came up?
9. Would you be receptive to using the internet or telephone to communicate with your keyworker?
10. Did you have a choice of key worker?
11. What skills does a key worker require?
12. How can the role best be utilised for people living with dementia/carers such as yourself?
13. Were there any gaps in the service?
14. What are the benefits of having a key worker?
15. Are there any questions we have not asked that you think we should be asking?
